# Real-time PCR analysis of enteric pathogens from fecal samples of irritable bowel syndrome subjects

**DOI:** 10.1186/1757-4749-3-6

**Published:** 2011-04-26

**Authors:** Teemu Rinttilä, Anna Lyra, Lotta Krogius-Kurikka, Airi Palva

**Affiliations:** 1 Department of Veterinary Biosciences, Faculty of Veterinary Medicine, University of Helsinki, PO Box 66, FIN-00014, Helsinki, Finland; 2 Alimetrics Ltd, Koskelontie 19B, FIN-02920, Espoo, Finland; 3 Danisco Sweeteners, Health and Nutrition, Sokeritehtaantie 20, FIN-02460, Kantvik, Finland

## Abstract

**Background:**

Growing amount of scientific evidence suggests that microbes are involved in the pathophysiology of irritable bowel syndrome (IBS). The predominant fecal microbiota composition of IBS subjects has been widely studied with DNA-based techniques but less research has been focused on the intestinal pathogens in this disorder. Here, we optimized a highly sensitive panel of 12 quantitative real-time PCR (qPCR) assays to shed light on the putative presence of intestinal pathogens in IBS sufferers. The panel was used to screen fecal samples from 96 IBS subjects and 23 healthy controls.

**Results:**

Fifteen IBS samples (17%) tested positive for *Staphylococcus aureus *with a thermonuclease (*nuc*) gene-targeting qPCR assay, whereas none of the healthy controls were positive for *S*. *aureus *(*p <*0.05). The *S. aureus *-positive IBS samples were confirmed by sequencing of the PCR amplicons. *Clostridium perfringens *was detected from IBS and control groups with a similar frequency (13% and 17%, respectively) with α-toxin (*plc*) gene -targeting qPCR assay while none of the samples tested positive for the *Cl. perfringens *enterotoxin-encoding gene (*cpe*).

**Conclusions:**

The qPCR panel consisting of 12 assays for an extensive set of pathogenic microorganisms provides an efficient alternative to the conventional detection of gastrointestinal pathogens and could accelerate the initiation of targeted antibiotic therapy reducing the risk of post-infectious IBS (PI-IBS). *S. aureus *has not been previously reported to be associated with the onset of IBS. Although we discovered significant differences in the prevalence of *S. aureus *between the study groups, its importance in giving rise to IBS symptoms requires further studies.

## Background

Irritable bowel syndrome (IBS) is a common multifactorial functional intestinal disorder of unknown etiology [1]. It is considered a major cause of abdominal discomfort and gut dysfunction worldwide with an estimated prevalence of 10% to 20% of the adult population, which makes it the most frequent diagnosis in gastroenterology [1-3]. Although not life-threatening, IBS is a major global health problem resulting in significant sensation of illness, poor quality of life, a high rate of work absenteeism and considerable health costs [4,5]. IBS is characterized by a variable combination of chronic and recurrent symptoms including abdominal pain or discomfort, irregular bowel movements, flatulence, and constipation or diarrhea [6]. According to the stool consistency, IBS subjects can be divided into three subcategories predominant in diarrhea (IBS-D), constipation (IBS-C) or alternating constipation and diarrhea *i.e*. the mixed subtype (IBS-M) [1,4,6].

The mechanisms of pathogenesis behind IBS are only partly understood and thus cannot be traced to a single organic factor. Instead, IBS is considered a complex biopsychosocial condition in which a multitude of mechanisms at the central and peripheral level interact [7]. The proposed mechanisms contributing to the etiology of IBS symptoms include visceral hypersensitivity, abnormal motor function, low-grade mucosal inflammation, food intolerance, altered gut microbiota as well as psychosocial and genetic factors [8-10]. However, it is often difficult to differentiate between the causes and effects, especially for chronic impaired states of health [11].

The role of enteric infections in the pathogenesis of IBS has been recognized for years and the development of IBS in subjects who have undergone a previous episode of infectious gastroenteritis has been reported in several prospective studies [12-18]. Infectious diarrhea due to *Escherichia coli *0157:H7, *Salmonella*, *Shigella *or *Campylobacter *has been shown to precede the development of post-infectious IBS (PI-IBS) [15,19,17,21]. However, there have been no reports regarding the involvement of other potentially pathogenic microorganisms on the etiology of the IBS. Furthermore, in PI-IBS the acute phase of the gastroenteritis has passed and the expected level of enteric pathogens could be low and thus difficult to detect. It is noteworthy that even low levels of enteric pathogen could imply the plausible causative agent in PI-IBS and contribute to the prolonged gastric symptoms. Traditionally clinical diagnostics of enteric infections have relied on conventional culturing techniques, which lack the potential for convenient large-scale diagnostics and the adequate accuracy and sensitivity necessary for low-level sub-clinical detection.

Molecular techniques have expanded our understanding regarding the microbial ecology of the gastrointestinal tract [22]. Quantitative real-time polymerase chain reaction (qPCR) is a powerful advancement of the conventional end-point PCR, which enables quantification of the target nucleic acid and reduces significantly the risk of 'carry-over' contamination [23]. Generally, the technique has exceptional performance characteristics including a wide dynamic range (up to 7 orders of magnitude) and high sensitivity. Therefore, it is a superior method for the accurate and targeted quantification of specific bacterial species or groups within microbial populations. The successful employment of qPCR with 16S rRNA gene -targeted primers for evaluating commensal intestinal bacterial species, groups or genera from fecal samples of IBS subjects has been reported previously by our group [24-29]. Here, we investigated the presence and abundance of selected intestinal pathogens in fecal samples of IBS subjects and healthy controls to shed light on the putative role of these bacteria in the pathogenesis of IBS. We applied a panel of 12 qPCR assays with SYBR Green I-chemistry targeting the virulence-associated or 16S rRNA genes and demonstrated that the qPCR approach has an advantage of being a highly sensitive and efficient technique for an extensive quantitative screening of intestinal pathogenic microorganisms from clinical samples.

## Methods

### Study subjects

A total of 96 IBS subjects fulfilling the Rome I [30] or II [6] criteria were recruited by experienced physicians (69 females and 27 males; age 20-73 years; mean 47 years). The subjects had originally been recruited for two independent studies and thus the inclusion and exclusion criteria were not identical. The IBS group consisted of subjects with IBS-D or IBS-M (81 subjects) and IBS-C (15 subjects) (Table 1). Due to two independent sample sets with different classification criteria, the IBS-D and IBS-M subgroups were combined for this study. All subjects fulfilling the Rome I criteria (n = 44; 33 females and 11 males) were 20-73 years of age and their general condition was confirmed as good by medical experts. Exclusion criteria for participation included presence of organic GI diseases, inadequately treated hypertension or pharmaceutically treated diabetes. Use of statins, pharmaceutically treated hypertension or coronary artery disease were not considered exclusion criteria if medication had been used for at least six months prior to the study with no changes in dosage. The IBS subjects fulfilling the Rome II criteria (n = 52; 36 females and 16 males) were 20-65 years of age and had undergone clinical investigation and endoscopy or barium enema of the gastrointestinal tract 0 to 1 years prior to the study. Exclusion criteria were pregnancy, lactation, organic intestinal diseases or other severe systematic diseases, previous major abdominal surgery, severe endometriosis and dementia. Patients that had received antimicrobial medication during the previous two months before the initiation of the study were also excluded whereas subjects with lactose intolerance were included if they reported to follow a low-lactose or lactose-free diet. A healthy control group (n = 23; 16 females and 7 males) (Table 1) devoid of gastrointestinal symptoms was recruited to form a control group for the IBS subjects. Intestinal disturbances (including lactose intolerance and celiac disease) and ongoing antibiotic treatments were considered exclusion criteria for the control subjects. The fecal samples of the IBS subjects or healthy controls described above have been analyzed previously for other microbial species or groups [24-28,31-33].

**Table 1:** Characteristics of the IBS subjects and healthy controls.

	IBS-D & IBS-M	IBS-C	Controls
Number of subjects	81	15	23
Average age (range)	47 (20-73)	49 (24-65)	45 (26-64)
Gender (female/male)	57/24	12/3	16/7
Predominant bowel habit	Diarrhea or mixed type	Constipation	-

The protocol was approved by the Human Ethics Committee at the Joint Authority for the Hospital District of Helsinki and Uusimaa (HUS), Finland for Rome II fulfilling IBS subjects, Kuopio University Hospital Ethical Committee for research, Finland for Rome I fulfilling IBS subjects and by the ethical committee of VTT, Finland for healthy controls. A written informed consent was acquired from each participating subject. Participation in the study was voluntary and the subjects were allowed to withdraw at any point without an explanation.

### Bacterial strains and culture conditions

The pathogenic bacterial strains used as positive and negative controls in this study included *Aeromonas hydrophila *(R5-11-1), *Bacillus cereus *(ATCC 9139), *Campylobacter jejuni *(Neqas 6037), *Clostridium difficile *(ATCC 9689), *Clostridium perfringens *(ATCC 13214), *Escherichia coli *EHEC O157:H7 (ATCC 43894), *Helicobacter pylori *(DSM 4867), *Listeria monocytogenes *(R14-2-1), *Salmonella enterica *serovar Infantis (R5-2-1), *Staphylococcus aureus *(ATCC 25923), *Yersinia enterocolitica *(R5-9-1) and *Yersinia pseudotuberculosis *(R5-9-4). *E. coli *was grown in Luria broth (Difco, USA) at 37C with shaking (220 r.p.m) and *A. hydrophila*, *B*. *cereus, L. monocytogenes, S*. Infantis, *S. aureus *and *Yersinia *spp. strains were cultured on blood agar plates at 37°C. *Cl. difficile *and *Cl. perfringens *were cultured at 37°C in fastidious anaerobe broth (LabM, UK) supplemented with 1% glucose using anaerobic incubation (Concept Plus Anaerobe Workstation, Ruskinn Technology, UK). *C. jejuni *and *H. pylori *were cultivated under microaerobic conditions at 37°C, using microaerobic atmosphere-generating system (CampyGen; Oxoid, UK). *C. jejuni *was cultured in *Campylobacter *enrichment broth (LabM) supplemented with 5% saponin-lysed horse blood and *H. pylori *was grown on blood agar plates.

### Fecal sample collection and DNA extraction

The fecal samples for bacterial studies were collected from each study subject and stored in sterile normal atmosphere containers or anaerobic containers at -80°C prior to analysis. The chromosomal DNA was isolated from fecal samples of the study participants as well as from individual bacterial reference strains. For fecal samples, the removal of undigested particles by washing the samples with repeated low-speed centrifugation, collection of the bacteria in the pooled supernatants with high-speed centrifugation and extraction of chromosomal DNA were performed essentially as described by Apajalahti et al. [34]. The DNA extraction of the individual pure culture reference strains was carried by applying the bead beating cell disruption technique. Briefly, the overnight cultured bacterial cells were pelleted by centrifugation in the presence of glass beads (Ø = 0.1 mm, Edmund Bühler, Germany). Subsequently, the cells were disrupted by 60 s cell mill homogenization (Bühler Vibrogen Cell Mill VL4, Edmund Bühler, Germany) and genomic DNA was purified from the homogenates by phenol-chlorophorm extraction and ethanol precipitation. Finally, the DNA concentrations of both fecal DNA preparations and pure culture bacterial genomic DNA were determined by a fluorometric method based on the *bis*-benzimide fluorochrome Hoechst 33258 (Bio-Rad, USA), which fluorescence when bound to double-stranded DNA. The measurements were performed with a Versafluor fluorometer (Bio-Rad, USA) with an excitation wavelength of 360 nm and emission wavelength of 460 nm.

### Real-time PCR assays and conditions

The optimized qPCR assay conditions and their oligonucleotide sequences applied in this study are summarized in Table 2. Briefly, the *Salmonella *spp. and *Aeromonas *spp. assays were set to amplify bacterial targets from the mentioned genera. The *Bacillus cereus *group assay included *B. cereus*, *B. thuringiensis*, *B. anthracis*, *B. mycoides*, *B. pseudomycoides*, *B. weihenstephanensis*, which share a high degree of chromosomal sequence similarity within the 16S rRNA gene. The *E. coli *assay targeted both enterohemorrhagic (EHEC) and enteropathogenic (EPEC) strains. The remaining assays were designed to be species-specific. The specificity of the primer pairs have been validated in earlier publications (see references listed in Table 2). The oligonucleotide primers were synthesized commercially by MWG-Biotech AG, Germany.

**Table 2:** Real-time PCR assays of the pathogen panel applied in this study.

PCR assay (Target gene)	Sequence (5'-> 3')	Amplicon size (bp)	T_a_ (°C)	MgCl_2_ (mM)	Reference
*Aeromonas *spp. (*aerA*)	F: GAGAAGGTGACCACCAAGAACA R: AACTGACATCGGCCTTGAACTC	232	61	2	[57]
*Bacillus cereus *group (16S rRNA)	F: TCGAAATTGAAAGGCGGC R: GGTGCCAGCTTATTCAAC	288	64	3	[58]
*Campylobacter jejuni* (*hipO*)	F: GACTTCGTGCAGATATGGATGCTT R: GCTATAACTATCCGAAGAAGCCATCA	344	58	3	[59]
*Clostridium difficile* (16S rRNA)	F: TTGAGCGATTTACTTCGGTAAAGA R: CCATCCTGTACTGGCTCACCT	157	58	3	[55]
*Clostridium perfringens* (*plc*)	F: AAGTTACCTTTGCTGCATAATCCC R: ATAGATACTCCATATCATCCTGCT	283	61	4	[60]
*Clostridium perfringens* (*cpe*)	F: GGTTCATTAATTGAAACTGGTG R: AACGCCAATCATATAAATTACAGC	154	58	3	[61]
EHEC/EPEC (e*aeA*)	F: ATGCTTAGTGCTGGTTTAGG R: GCCTTCATCATTTCGCTTTC	248	65	4	[62]
*Helicobacter pylori* (16S rRNA)	F: GAAGATAATGACGGTATCTAAC R: ATTTCACACCTGACTGACTAT	139	58	4	[55]
*Listeria monocytogenes* (*iap*)	F: CTAAAGCGGGAATCTCCCTT R: CCATTGTCTTGCGCGTTAAT	174	61	4	[63]
*Salmonella *spp. (*invA*)	F: GTGAAATTATCGCCACGTTCGGGCAA R: TCATCGCACCGTCAAAGGAACC	281	64	2	[64]
*Staphylococcus aureus* (*nuc*)	F: GCGATTGATGGTGATACGGTT R: AGCCAAGCCTTGACGAACTAAAGC	279	60	3	[65]
*Yersinia enterocolitica* (*ail*)	F: GGTCATGGTGATGTTGATTACTATTCA R: CGGCCCCCAGTAATCCATAA	90	58	4	[66]

The qPCR analyses were performed with the iCycler iQ Real-Time PCR Detection System (Bio-Rad, USA) using iCycler IQ 96-well optical grade PCR plates (Bio-Rad, USA) covered with optical-quality sealing film (Bio-Rad, USA). The optimal annealing temperatures and MgCl_2 _concentrations (2.0 mM, 3.0 mM, 4.0 mM or 5.0 mM) were determined for each assay. The amplification reactions were carried out in triplicate in a volume of 25 μl with 1:75 000 dilution of SYBR Green I (Molecular Probes, USA), 10 mM Tris-HCl (pH 8.8), 150 mM KCl, 0.1% Triton X-100, 2-5 mM MgCl_2_, 100 μM each dNTP, 0.5 μM each primer, 0.6 U Dynazyme II polymerase (Finnzymes, Finland) and either 5 μl of template, negative control comprising an extensive set of representative gastrointestinal bacterial species or deionized sterile water as no-template control (NTC). The amplification involved one cycle at 95°C for 5 min for initial denaturation followed by 40 cycles of denaturation at 95°C for 15 s, primer annealing at 50-70°C for 20 s, extension at 72°C for 30 s and an additional incubation step at 80-85°C for 30 s to collect the fluorescent data. All clinical DNA samples of IBS subjects and healthy controls were analyzed in randomized order on several qPCR plates.

Tenfold dilution series of 0.1 pg to 10 ng (corresponding approximately to 30 - 100 to 3.0 × 10^6 ^- 1.0 × 10^7 ^genomic equivalents) of target species genomic DNA were used as standards in conjunction with 25 ng and 250 ng of each fecal DNA preparation in the same qPCR run. The following genome sizes for the standard strains were applied: 4.5 Mb for *Aeromonas *spp., 5.4 Mb for *B. cereus group*, 1.6 Mb for *C. jejuni*, 4.0 Mb for *Cl. difficile*, 3.2 Mb for *Cl*. *perfringens*, 5.4 Mb for EHEC and EPEC, 1.7 Mb for *H. pylori*, 2.9 Mb for *L. monocytogenes*, 4.8 Mb for *Salmonella *spp., 3.5 Mb for *S. aureus *and 4.8 Mb for *Y. enterocolitica*.

To determine specificity of the PCR reactions, a melt curve analysis was carried out in conjunction with each amplification runs by slow cooling from 95°C to 60°C, with fluorescence collection at 0.3°C intervals and a hold of 10 s at each decrement. For the verification of correct product size, the PCR amplicons were stained with ethidium bromide and subjected to electrophoresis on 2% agarose gels.

### Sequencing of *Staphylococcus aureus *PCR products

In addition to qPCR targeting *S. aureus*, sequencing of the PCR amplicons (approximately 279 bp) was carried out to verify the identity of the obtained gene fragments. The PCR products were purified with the QIAquick Gel Extraction Kit (Qiagen, Germany) after excising the DNA fragments from a 1.25% SeaPlaque agarose gel (Cambrex, USA), and eluted in 35 μl of elution buffer. The concentration of the purified amplicons was estimated with serially diluted samples on 0.8% agarose gels with ethidium bromide staining. The sequencing was carried out with an ABI 310 Genetic Analyzer (Applied Biosystems, USA) using the Big Dye Terminator chemistry (Applied Biosystems, USA). Two sequencing reactions were performed from individual PCR products with the *S. aureus nuc *gene-specific forward and reverse primers *i.e*. each amplicon was sequenced on both strands. Finally, the sequences were processed with Sequencher™ 3.0 sequence analysis software (Gene Codes Corporation, USA) and matched to existing GenBank sequences by using the nucleotide-nucleotide Basic Local Alignment Search Tool (BLAST) analysis tool of NCBI National Center for Biotechnology Information (NCBI) [35].

### Data analysis

In qPCR analyses, the numbers of target microbes remained below the detection limit of respective assays in the majority of samples. These values may not be truly zero or missing values, but are caused by technical limitations of the qPCR technique. Therefore, for data analysis, the undetected samples were given a value, which corresponded to the limit of detection of the respective qPCR assay. The raw bacterial qPCR data were transformed to log_10 _ratios of the number of target genomes in one gram of feces.

For statistical comparison, Pearson's chi square test was performed to obtain correlations between the presence/absence results of pathogen detection by qPCR and the different subject groups with Stata 9.2 Data Analysis and Statistical Software. The *p*-value of the significance probability less than or equal to 0.05 was considered statistically significant. In addition, the R software environment for statistical computing and graphics [36] was applied with R-scripts for data illustration.

## Results

### Optimization of the SYBR Green I-based real-time PCR assays

Quantitative real-time PCR procedures, which utilized 12 previously designed oligonucleotide primer pairs for the detection of an extensive set of intestinal pathogens, were optimized for the application in our qPCR quantification system with SYBR Green I-chemistry. The rationale for the re-optimization was the fact that majority of the assays were originally developed for conventional end-point PCR applications. The primer sequences, targets organisms, PCR product sizes as well as optimal annealing temperatures and MgCl_2 _concentrations are summarized and listed in Table 2. Although the specificity of the primer pairs has been previously verified, the potential cross-reactivity of the oligonucleotides was evaluated using BLAST search of GenBank [35]. For the 16S rRNA-gene-targeted assays, the specificity was verified also by submitting the primer sequences to Probe Match program provided by the Ribosomal Database Project (RDP II) [37]. In the *in silico *analyses, no cross-reaction with closely related or undesired bacterial species was observed with any of the primer pairs tested.

A dynamic range of five logs from 0.1 - 1 pg to 10 ng of specific target genome, which corresponds to approximately 30 - 100 to 3.0 × 10^6 ^- 1.0 × 10^7 ^genomic equivalents per qPCR reaction was obtained with seven out of eight assays when serially diluted chromosomal DNA was used as template. The exception was *B. cereus *group assay with the dynamic range of four logs. The lowered sensitivity of this assay was due to excessive formation of primer-dimers, which hampered the amplification of smallest 0.1 pg standards. The sufficient upper limit of the standard curve was not determined and could actually be higher further increasing the dynamic range of the applied assays. In addition, no signals above the detection limit were obtained from negative controls with any of the tested assays.

### Real-time PCR analysis of pathogens from fecal DNA samples

A total of 96 IBS and 23 control fecal specimens were subjected to qPCR analyses with newly optimized assays targeting a wide variety of intestinal pathogenic bacteria. The IBS subjects were divided according to their symptoms into constipation-predominant IBS and diarrhea-predominant or mixed-type IBS subgroups. In the analyses of *Aeromonas *spp., *B. cereus *group, EHEC and EPEC, *L. monocytogenes*, *Salmonella *spp. and *Y. enterocolitica *all samples from IBS and control subjects remained below the limit of detection (~10^4 ^bacterial genomic equivalents per gram of sample) of the respective qPCR analyses. Therefore, with these bacterial species the qPCR assays could only be tested with the reference strains. Conversely, in the analysis of *S. aureus, Cl. perfringens, H. pylori *and *Campylobacter *spp. positive signals were obtained.

**Figure 1:**
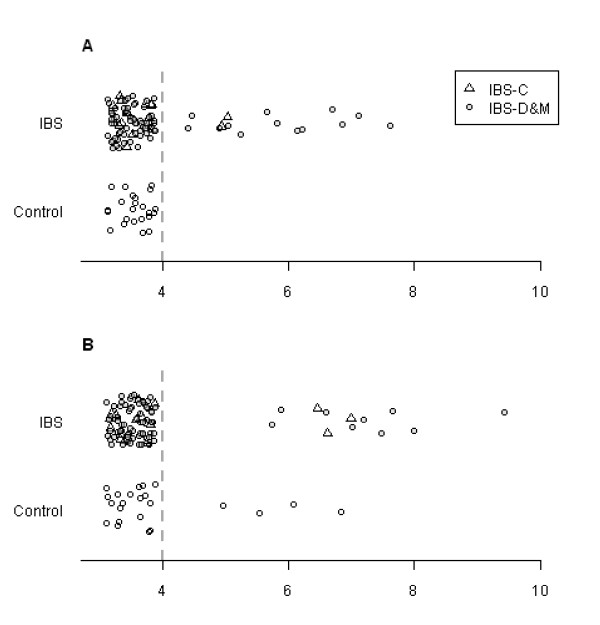
**Real-time PCR results of the assays for *Staphylococcus aureus nuc *gene (A) and *Clostridium perfringens plc *gene (B)**. The values are target genomes per gram of fecal sample (log_10 _values). The detection limit is set to 10^4 ^bacterial genomes. The abbreviations: IBS-C and IBS-D&M stand for constipation-predominant irritable bowel syndrome and diarrhea-predominant or mixed-type irritable bowel syndrome, respectively.

The qPCR analysis of *S. aureus *revealed that 15 IBS samples (prevalence of 17%) amplified within the linear range of the standard curve giving a positive result with the quantities ranging from 2.5 × 10^4 ^to 4.0 × 10^7 ^genomic equivalents per gram of feces (Figure 1A). The positive signals were initially verified by melting curve analysis (T_m _= 83.5°C) and agarose gel electrophoresis. On the contrary, no positive *S. aureus *signals were obtained with the samples from healthy controls. This result was statistically significant at the risk level of *p *< 0.05 (Pearson's Chi-square test). The *S. aureus*-positive IBS samples were relatively evenly distributed among the two subcategories as the frequencies of positive samples in IBS-D&M and IBS-C subgroups were 16% and 13%, respectively. No clear relation to age or gender was observed among the *S. aureus *positive samples.

In a screening process of fecal specimens with a qPCR assay targeting the α-toxin encoding the *plc *gene of *Cl. perfringens*, 12 samples of IBS subjects were positive with the quantities ranging from 5.6 × 10^5 ^to 2.7 × 10^9 ^genomic equivalents per gram of feces (Figure 1B). The obtained signals were verified to be true positives by melting curve analysis (T_m _= 83.5°C) and agarose gel electrophoresis, which both resulted in identical results with the reference strain. The highest frequency of positive samples was observed in the IBS-C subgroup with a prevalence of 20% while in the IBS-D&M subgroup the prevalence of *Cl. perfringens*-positive samples was 11%. The average age of the subjects yielding a *Cl. perfringens *positive signal was 50.5, which is slightly higher as compared to the average age of the IBS subjects. It is noteworthy that three of the *Cl. Perfringens *-positive samples from IBS-D&M subgroup were also discovered to be positive in the analysis of *S. aureus*.

In addition to 12 IBS subjects, fecal samples of three males and one female of the healthy control group possessed the *plc *gene-positive *Cl. perfringens *corresponding to the prevalence of 17%. The quantities ranged from 9.0 × 10^4 ^to 6.9 × 10^6 ^genomic equivalents per gram of fecal sample (Figure 1B). Due to the relatively equal prevalence of *Cl. perfringens *in the different subject groups, no statistically significant differences with the Pearson's Chi-square test were observed. In addition to the α-toxin gene-targeted real-time PCR, all samples were screened for *cpe*, which is a gene encoding enterotoxin (CPE), a causative agent of *Cl*. *perfringens *Type A food poisoning. No indications of the presence of the *cpe *gene possessing *Cl. perfringens *were observed in either of the IBS subgroups or healthy controls.

The occurrence of *H. pylori *was detected in the samples of three IBS subjects with the average age of 57 years whereas no positive cases were observed among control subjects. The *H. pylori *numbers ranged from 8.3 × 10^5 ^to 1.0 × 10^7 ^genomic equivalents per gram of fecal specimen. *C*. *jejuni *was detected only in one subject belonging to the IBS-D&M subgroup.

### *Staphylococcus aureus *PCR product verification

The samples yielding a *S. aureus *signal were confirmed by sequencing the PCR amplicons and by performing a BLAST database search against the GenBank sequences. All 15 sequences were found to be 100% identical and shared complete sequence similarity with the thermonuclease precursor (*nuc*) gene of *S. aureus *subsp. *aureus *in the GenBank database. Hence, based on the obtained sequencing results in conjunction with the correct melting curve profile (T_m _= 83.5°C) of the amplicons, the obtained findings were regarded as 'true positives' of *S. aureus*.

## Discussion

A comprehensive investigation of the gut microbiota is fundamental for the understanding of the role of bacteria in IBS. Therefore, predominant enteric bacteria in the fecal samples of IBS subjects have been widely studied with different DNA-based techniques [24,29,38-40]. To our knowledge, however, there are relatively few reports focusing on the detection of intestinal pathogens, the abundance of which is likely to be significantly lower as compared to dominant microorganisms present in the gut. Moreover, as PI-IBS is considered a prolonged state of functional gastrointestinal symptom triggered by an acute gastroenteritis, the level of the original causative agent may be much lower or even undetectable after the acute phase of an enteric infection has passed. Thus, much of the data regarding the role of pathogens as causative agents of IBS is likely to be limited since the routine clinical investigations commonly rely on bacterial culturing techniques possibly lacking the sufficient sensitivity for detecting low levels of pathogens from fecal specimens. In this study, our aim was to discover whether the selected intestinal pathogenic bacterial species or groups are associated with IBS in comparison with healthy controls even at low detectable levels by applying a highly sensitive qPCR approach with the primer pairs targeted either to a virulence gene or the 16S rRNA gene.

An extensive qPCR screening of fecal DNA samples revealed that *S. aureus *was detected in IBS subjects with a frequency (17%) that differed from that of the control group with a statistical significance. *S. aureus *is a food-borne pathogen known to cause food poisoning but it is difficult to obtain accurate estimates of the precise incidence of *S. aureus *intoxications since many cases are not specified or reported. The disease occurs as a result of the ingestion of foods or beverages containing one or more preformed enterotoxins (SE) produced by the species and it is characterized by symptoms including diarrhea, nausea and abdominal cramps [41]. Classically, SEs have been divided into five major serological types (SEA, SEB, SEC, SED, and SEE) on the basis of their antigenic properties [42] but during the last decade, nine new types have been discovered (SEG to SEO). In this study, we targeted the *nuc *gene as a common *S. aureus *marker in the real-time PCR analyses. Therefore, the occurrence of enterotoxin producing strains within the *S. aureus *positive samples detected remains to be evaluated. It is noteworthy, however, that in a study by Pinto and coworkers [43], a total of 40 out of 131 food isolates analyzed were positive for the *se *genes (prevalence of 31%), the *sec *genotype being the most frequent.

Thus far, *S. aureus *has not been reported to be associated with the onset of IBS although there is evidence that IBS can be triggered by an episode of acute gastroenteritis in approximately 15% of subjects (for review see Smith and Bayles [44] as well as Spiller and Garsed [11]). The common bacteria, which have been associated with PI-IBS include the foodborne pathogens *C*. *jejuni*, *E. coli *O157:H7 and *Salmonella *spp. [13,15,19,45,46]. In this set of fecal samples, one IBS subject was *C. jejuni *-positive but no signals of *Salmonella *or enteropathogenic and enterohaemorrhagic *E. coli *were observed. The association between *H. pylori *infection and IBS subjects has been reported by Su et al. [47]. They discovered that 33 of the 69 subjects included in the study (a prevalence of 47.8%) had *H. pylori *infection, which was associated with functional dyspepsia. In our qPCR analysis, *H. pylori *was detected in three IBS subjects one of which harbored *H. pylori *at relatively high level (~10^7 ^genomic equivalents per gram of feces; data not shown). This IBS subject had been previously treated for *H. pylori *but the treatment period was not fully completed. It is worth mentioning that the most common habitat of *H*. *pylori *is the human stomach and the beginning of the upper digestive tract. Here, all the analyses were carried out on fecal samples, which may explain the lack of positive cases. Although previous gastroenteritis was not an inclusion criterion for the IBS subjects in this study, the obtained results in conjunction with earlier findings by other researches suggests that a number of different gastroenteritis-inducing bacteria may be involved in IBS. Hence, it is indeed likely that the inflammatory response of the host rather than a particular individual pathogenic species is probably the key factor triggering the onset of IBS [48]. It remains unclear, however, whether the different pathogenic species pose an equal risk of developing the disorder.

*Cl. perfringens *was detected from IBS and control groups with a similar frequency when targeting the gene encoding α-toxin (*plc*). This result along with the fact that *Cl. perfringens *is a commensal species in the stool of some normal healthy subjects [49] implies that there is no association between *Cl. perfringens *and the pathogenesis of the IBS at least within the subjects of this study. Furthermore, the detection *Cl. perfringens *enterotoxin in fecal samples has been suggested to be the definitive method of implicating this organism as the cause of diarrhea and abdominal cramps due to the gastroenteritis [50]. In this study, none of the samples tested positive for the chromosomally located *Cl. perfringens *enterotoxin-encoding gene (*cpe*).

Complex microbial communities and challenging sample matrix make DNA extraction from feces technically demanding. Fecal matter contains several inhibitory factors such as bile salts, haemoglobin degradation products and complex polysaccharides that can have significant adverse effects on the efficiency and sensitivity of PCR-based assays [51-53]. Fecal PCR inhibitors generally exert their effects by interacting with target DNA or blocking the enzyme activity of thermostable DNA polymerases [54]. Hence, the best way to avoid PCR inhibition is to prevent the inhibitor from being processed with the sample. Another prerequisite for a successful molecular analysis of complex microbiota is the efficiency of cell disruption step and subsequent recovery of bacterial DNA. It is apparent that if the isolated DNA does not accurately represent the microbial composition of the original sample due to inefficient cell lysis, the results obtained will be unreliable and biased. The good recovery rates of fecal bacterial DNA extraction method applied in this study [34] have been confirmed earlier by our research group [55]. The feasibility of the technique was also recently further demonstrated in a comparative study in which four widely used DNA extraction protocols were evaluated [56].

In this report, we introduced an optimized and validated panel of qPCR assays applicable for highly sensitive and specific high-throughput gastrointestinal pathogen detection from clinical samples. A total of 12 pairs of oligonucleotide primers for several common pathogenic species or groups were carefully selected from earlier publications. The specificity of these primers was reconfirmed by applying both *in silico *and *in vitro *analyses and the optimal qPCR conditions were determined. As a result, with this pathogen panel the target bacteria were detectable at a minimum concentration range of approximately 10^4 ^bacterial genomes per gram of fecal sample, which corresponds to the sensitivity to detect 0.000001% subpopulations of the total fecal microbiota.

## Conclusions

We applied the newly optimized panel for the screening of an extensive set of fecal DNA samples of IBS subjects in order to shed light on the putative role of pathogenic bacteria in IBS. Although significant differences in the prevalence of *S. aureus *between the study groups were observed, its importance in giving rise to IBS symptoms requires further studies. Nevertheless, the results obtained indeed support the earlier suggestions with regards to the role of intestinal pathogens in IBS.

Overall, the qPCR panel optimized and validated in this study provides a feasible and efficient alternative to the conventional detection of gastrointestinal pathogens and thus could accelerate the initiation of targeted antibiotic therapy reducing the risk of PI-IBS. However, the future challenge will be to fine-tune the analysis pipeline from the sample preparation to real-time PCR in order to obtain the results within in 3 to 4 hours. This would provide a valuable asset for such clinical microbiology laboratories, the diagnostics of which rely currently on the bacterial culturing techniques.

## Competing interests

The authors declare that they have no competing interests.

## Authors' contributions

TR participated in the design of the study, selected the primers applied, performed and supervised qPCR experiments, collected and analyzed data, and prepared the draft manuscript. AL participated in the design of the study, supervised the qPCR experiments, and helped to draft the manuscript. LK-K carried out qPCR experiments and helped to draft the manuscript. AP participated in the design of the project, coordinated and supervised the study, and helped to draft the manuscript. All authors approved the final manuscript.
